# Mutagenic effects induced by the attack of NO_2_ radical to the guanine-cytosine base pair

**DOI:** 10.3389/fchem.2015.00013

**Published:** 2015-03-06

**Authors:** José P. Cerón-Carrasco, Alberto Requena, José Zúñiga, Denis Jacquemin

**Affiliations:** ^1^Departamento de Química Física, Universidad de MurciaMurcia, Spain; ^2^Chimie et Interdisciplinarité, Synthèse, Analyse, Modélisation, UMR Centre National de la Recherche Scientifique, Université de NantesNantes, France; ^3^Institut Universitaire de FranceParis, France

**Keywords:** guanine–cytosine, NO_2_ radical, induced mutation, proton transfer reaction, tautomeric equilibria, rare tautomers, computational chemistry, density functional theory

## Abstract

We investigate the attack of the nitrogen dioxide radical (NO^•^_2_) to the guanine—cytosine (GC) base pair and the subsequent tautomeric reactions able to induce mutations, by means of density functional theory (DFT) calculations. The conducted simulations allow us to identify the most reactive sites of the GC base pair. Indeed, the computed relative energies demonstrate that the addition of the NO^•^_2_ radical to the C8 position of the guanine base forms to the most stable adduct. Although the initial adducts might evolve to non-canonical structures via inter-base hydrogen bonds rearrangements, the probability for the proton exchange to occur lies in the same range as that observed for undamaged DNA. As a result, tautomeric errors in NO_2_-attacked DNA arises at the same rate as in canonical DNA, with no macroscopic impact on the overall stability of DNA. The potential mutagenic effects of the GC–NO^•^_2_ radical adducts likely involve side reactions, e.g., the GC deprotonation to the solvent, rather than proton exchange between guanine and cytosine basis.

## 1. Introduction

Free radicals are naturally present in the biological medium as intermediates of the cellular metabolism. Although necessary to keep the normal biochemical activity, these highly reactive species tend to react, when present in excess, with a wide panel of biomolecules including DNA (Cadet et al., 2010). Accordingly, free-radical-induced DNA damage (mutations) might eventually initiate degenerative diseases, cardiovascular problems and cancers (von Sonntag, 2009). The reactive nitrogen oxide species (RNOS) family is a representative example of the double role played by free radicals (Lonkar and Dedon, 2011). Indeed, it has been established that mammalians cells endogenously produce RNOS as critical biological mediators (Ignarro, 1990). However, intracellular amounts of RNOS can overpass the tolerable limit by exposure to external chemical agents, such as cigarette smoke or air pollution (Pacher et al., 2007), and physical agents like high-energy radiations (Douki and Cadet, 2008).

**Figure 1:**
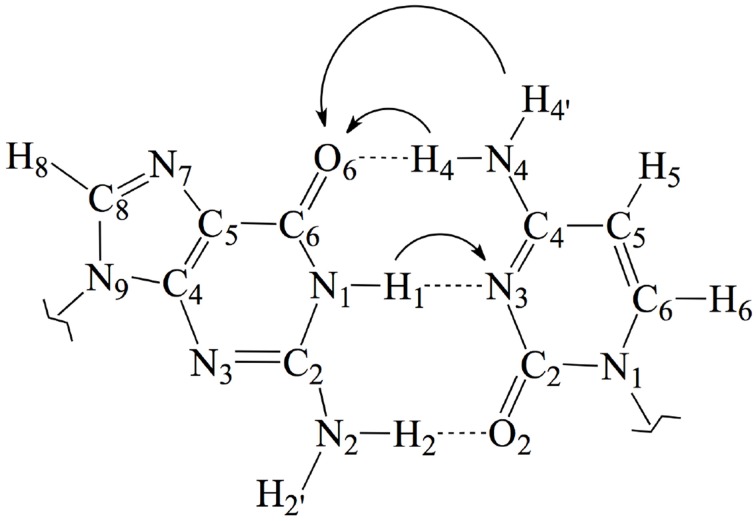
**Chemical structure and atomic numbering of the canonical GC base pair**. Both N9 in G and N1 in C are the connections to the lateral backbone in DNA. In an isolated GC base pair, these positions are occupied by two hydrogen atoms. Arrows show the possible displacements of the protons H1, H4, and H4′ during the tautomeric reactions.

One of the most important RNOS is the nitrogen dioxide radical, which is mainly present under its monomer NO^•^_2_ form in physiological conditions (Augusto et al., 2002). Like any free radical, NO^•^_2_ might react by electron transfer, hydrogen atom abstraction and/or radical addition to unsaturated bonds (Galano, 2007; Cerón-Carrasco et al., 2010). As for a reaction with DNA is concerned, the latter mechanism involves the addition of the radical to one of the unsaturated bonds of the guanine–cytosine (GC) base pair. There are, in particular, five possible reactive positions in the GC pair: C4, C5, and C8 in the guanine [C4(G), C5(G), and C8(G)] and C5 and C6 in the cytosine [C5(C) and C6(C)]. The atomic numbering is shown in Figure 1. Previous experimental and theoretical evidences indicated that C8(G) is the most reactive site for OH^•^ radical attack (Fortini et al., 2003; Shukla et al., 2004; Jena and Mishra, 2005; Shukla and Mishra, 2007; Zhang and Eriksson, 2007; Bergeron et al., 2010; Cerón-Carrasco and Jacquemin, 2012). Agnihotri and Mishra have also shown that NO^•^_2_ quickly reacts with the guanine radical cation (G^•+^) at this same position (Agnihotri and Mishra, 2009, 2010, 2011), which might lead to DNA damage through the formation of an intermediate 8-nitroguanine structure (Misiaszek et al., 2005). The reaction of NO^•^_2_ radical with the undamaged (neutral and closed-shell) GC pair has been, however, much less explored, so additional work is required to understand the biological action of such radical once inside the cellular medium.

In this work, we use an state-of-the-art theoretical approach within the density functional theory (DFT) framework to investigate the possible mutagenic effects induced by the NO^•^_2_ radical. In particular, we study the formation of non-canonical Watson and Crick structures arising from activation of the tautomeric equilibria in the GC base pair. These equilibria involve proton transfer (PT) between the guanine and cytosine basis yielding the so-called rare tautomeric forms that have been proposed as a plausible source of genetic errors (Löwdin, 1963; Florián and Leszczyński, 1996; Dannenberg and Tomasz, 2000; Gorb et al., 2004; Kumar and Sevilla, 2010; Villani, 2010a; Jacquemin et al., 2014). In undamaged DNA, GC base pairs naturally exchange the position of the H1, H4 and H4′ protons (see Figure 1), eventually disrupting the standard hydrogen-bond pattern that maintain the double helix bounded and thus inducing genetic errors during the cell replication (Villani, 2010b). Consequently, the tautomeric equilibria can be altered by external agents facilitating PT reactions (Khanduri et al., 2011). The main goal of this contribution is therefore to determine whether NO^•^_2_ radical contributes to the mutagenic processes by shifting PT mechanism toward rare-tautomeric GC structures.

## 2. Methods

**Figure 2:**
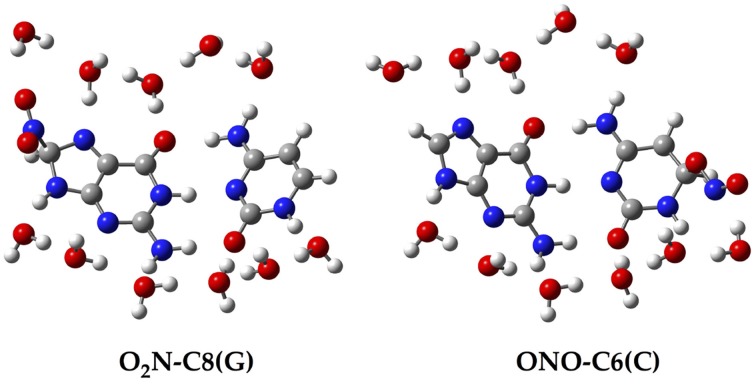
**Chemical structure for two of the single GC–radical adduct models: O_2_N–C8(G) and ONO–C6(C)**.

Since all the structures under study correspond to radical forms, the bullet superscript is hereafter omitted in the text in order to lighten the notation. We employ two different chemical models. In an early stage, a single GC base pair is used to determine the relative stabilities of all possible GC–radical adducts. More specifically, the NO_2_ radical is added to all possible reactive sites of the GC base pair through the two possible binding modes, e.g., ONO–CX and O_2_N–CX, where CX stands for the attacked carbons: C4(G), C5(G), C8(G), C5(C), and C6(C). The resulting adducts are next fully solvated with eleven water molecules, as illustrated in Figure 2, in order to mimic the solvent effects of a single solvated GC pair (Kumar et al., 2008). All these structures are optimized without symmetry constraints at the M06-2X/6-311G(d,p) level (Zhao and Truhlar, 2008a,b). The vibrational frequencies are computed at the same level of theory to confirm the absence of imaginary frequencies so that real minima are obtained. The total energies of all stationary points are then improved using the same exchange-correlation functional with the more extended atomic basis set, 6-311++G(d,p). Also, the well-known polarisable continuum model (PCM) is additionally used to account for solvent effects beyond the explicitly-treated first hydration shell (Tomasi et al., 1999, 2005).

**Figure 3:**
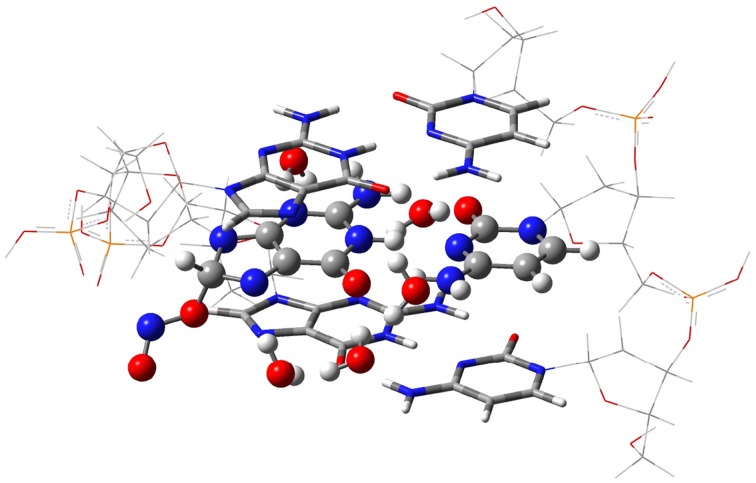
**The ONIOM model for the DNA-embedded GC-radical adduct**. The high layer for the ONO-C8(G) adduct is represented as ball and sticks whereas the confining base pair of the medium layer are shown as tube, and the lateral back-bone placed in the low layer are displayed as wireframe.

The isolated GC–radical adducts might form indeed over-distorted structures compared to real DNA, since the stacking effect is neglected. Consequently, the single base pair model is next refined by sandwiching the most stable adduct into at double-stranded B-form trimer d(5′-GGG-3′)d(3′-CCC-5′), as shown in Figure 3. This model includes both nucleotides moieties and sugar-phosphate backbone chains (Chen et al., 2009, 2011; Cerón-Carrasco et al., 2011). Since phosphates are negatively charged at physiological pH, the neutrality of the systems can be ensured by either adding counterions, e.g., Na^+^ or K^+^, or protonating the phosphate groups. Since the backbone does not significantly affect PT reactions in DNA (Close and Øhman, 2008; Chen et al., 2009), we go for the latter procedure to have a neutral system and consequently facilitate the convergence of the calculations. The GC-radical adduct is therefore located between two GC base pairs, which correctly mimic the π-stacking interactions in real DNA (Chen et al., 2009). We should underline that the stacked DNA model includes five water molecules in the vicinity of N3(G), O6(G), N7(G), O2(C), and N4(C) atoms (see atomic numbering in 1), and not eleven as in the single GC–radical adduct. These five sites correspond to the solvent exposed heteroatoms in DNA, so five water molecules are enough to provide a suitable representation of the hydrogen-bonds network formed between the base pair and the first hydration shell of the stacked structure (Schneider and Berman, 1995; Auffinger and Westhof, 2000; Makarov et al., 2002).

Due to the size of the DNA fragment (ca. 200 atoms), we use the hybrid ONIOM approach (Dapprich et al., 1999), which allows us to split the system into different regions, called layers. In this system, the layer of interest (defined as high layer) is the central GC–NO_2_ radical adduct plus the five explicit water molecules. This layer is treated at the same level that the isolated model, i.e., by using M06-2X/6-311G(d,p) for the optimization and PCM-M06-2X/6-31++G(d,p) for single-point energy calculations (Vreven et al., 2001; Mo et al., 2004). The two border GC base pairs that confine the central GC-radical adduct form the medium layer, which is described at the less demanding M06-2X/6-31G(d) level, while the lateral sugar-phosphate backbone (low layer) is simulated with the semiempirical PM6 method (Stewart, 2007). In this computation protocol, only the high layer is optimized whereas the rest of the system is frozen in the space. Such partial optimization procedure retains the characteristic double helix form of DNA while allowing relaxation of the central base pairs during the proton transfer reaction. Analytic calculations of the vibrational frequencies confirm the nature of the obtained structures to be minima (no imaginary frequencies) or transition states (a single imaginary frequency corresponding to the stretching of the transferred protons). All calculations are carried out with Gaussian09 (Frisch et al., 2009).

## 3. Results and discussion

Although previous experimental and theoretical evidences suggest that the C8(G) is the preferred site for adding radicals to the GC base pair (Kumar et al., 2011), we have calculated the relative stabilities of all possible GC–radical adducts to provide an accurate picture of the NO_2_ radical reactivity. The relative energies obtained for the located adducts are listed in Table 1, with the reference value being taken as the most stable GC–radical adduct: ONO–C8(G). As observed, there are two main radical-attacking sites: C8(G) and C6(C). It wasn't possible to optimize GC-radical adduct at other positions, with the only exception of ONO–C5(C) at a significantly higher energy (11.76 kcal.mol^−1^). This finding is consistent with previous results obtained by Zhang and Eriksson using DFT calculations to determine the reactivity of the OH radical with the neutral closed-shell GC base pair (Zhang and Eriksson, 2007). These authors concluded that the two most stable binding sites for the GC–OH mutation are C8(G) and C6(C) (Zhang and Eriksson, 2007). In contrast, there are two possible binding modes (O_2_N– and ONO–) in the present case. Inspection of Table 1 reveals that the attack of the NO_2_ radical could proceed equally through the nitrogen or the oxygen atom. This is a remarkable result: the binding mode does not significantly affect the final stability of the generated adduct since the reported difference is negligible (<0.40 kcal.mol^−1^).

**Table 1:** **Relative energy (ΔE/kcal.mol^−1^), theoretical interbase bond distances (in Å) and Mulliken atomic charges (in |***e***|) for the single GC–radical adducts**.

**Adduct**	**ΔE**	**Distance**			**Charge**		
		**O_**6**_–N_**4**_**	**N_**1**_–N_**3**_**	**N_**2**_–O_**2**_**	**H_**4**_**	**H_**1**_**	**H_**2**_**
ONO–C8(G)	0.00	2.908	2.907	2.839	0.34	0.49	0.38
O_2_N–C8(G)	0.15	2.894	2.890	2.838	0.38	0.56	0.36
O_2_N–C6(C)	7.69	2.843	2.912	2.891	0.41	0.57	0.35
ONO–C6(C)	8.08	2.859	2.920	2.878	0.34	0.47	0.32
ONO–C5(C)	11.76	2.871	2.935	2.876	0.34	0.52	0.35

**Figure 4:**
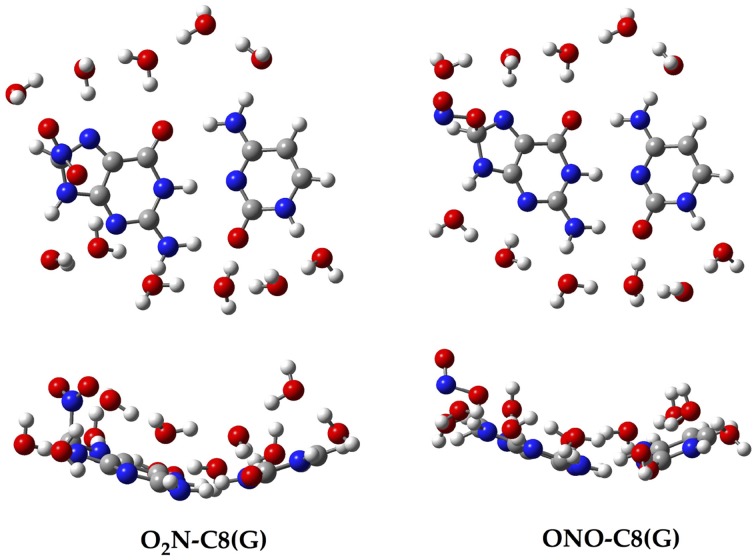
**Top and side views of the optimized structure for the O_2_N–C8(G) and ONO–C8(G) adducts**.

Aiming to characterize better the structural changes induced by the radical, we also include in Table 1 the interbase distances and the partial atomic charges of the protons involved in the intramolecular hydrogen bonds. Although there are slight differences (ca. 0.1 Å) in the interbase separation, adding the NO_2_ radical does significantly alter the GC geometry. The structures of the two most stable GC–radical adducts, namely O_2_N–C8(G) and ONO–C8(G), are shown in Figure 4. The side views show a clear bent structure as a result of the radial addition. Such departure from planarity can be quantified through the dihedral angle formed by C6(G)C2(G)C2(C)C4(C) (see atomic numbering in Figure 1), which has a value of around 1° for undamaged GC, but close to 10° in both GC–radical adducts. This is a logical consequence of the selected model, which mimics the reactivity of a single GC base pair in solution but does not reproduce the DNA environment, and in particular the π-stacking constrain.

More intriguing are the results shown in Table 1 for the atomic charges. As observed, the O_2_N– biding mode results in larger (more positive) atomic charges for the transferred proton during the tautomeric reaction, e.g., H4 and H1, compared to its ONO– counterpart. Although limited, these changes might affect the stability of the induced mutation since the atomic charges are directly related to the proton acidity and, consequently, to the tendency of forming tautomers.

The single GC base pair model provides the first clues for the attack of the NO_2_ radical to DNA, but the more refined model is required for biological analysis because (i) bend structures imply energetic penalties once inside DNA due to the π-stacking interactions; and (ii) the location of the radical could be modified by the chemical environment of DNA, e.g., by the interaction with other base pairs and/or later sugar-phosphate backbone. Consequently, the two most stable adducts, O_2_N–C8(G) and ONO–C8(G), are “docked” into the DNA fragment shown in Figure 3. In contrast with the single GC model in which both binding modes are practically isoenergetic, the ONO–C8(G) adduct is now 3.23 kcal.mol^−1^ more stable than the O_2_N–C8(G) adduct in DNA, which confirms the importance of accounting for all key interactions.

**Figure 5:**
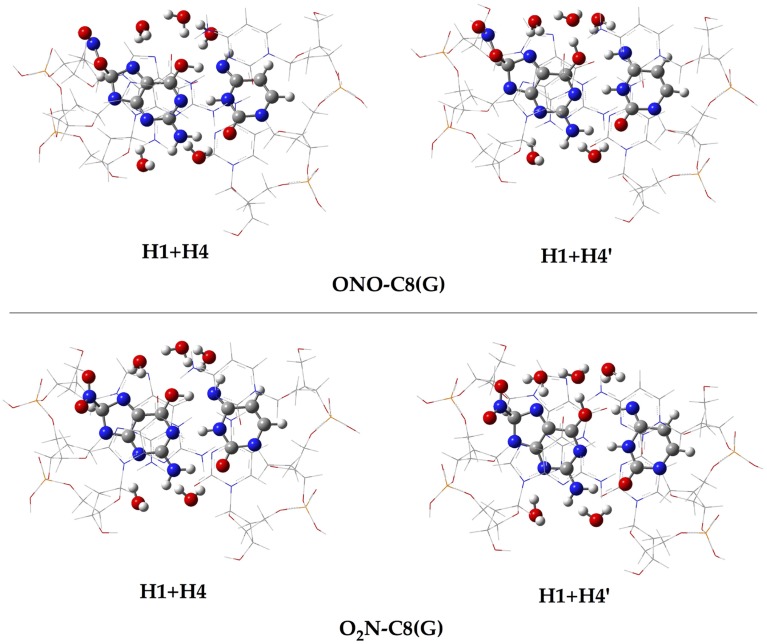
**Chemical structures of the canonical rare tautomeric form optimized for the O_2_N–C8(G) and ONO–C8(G) adducts: H1+H4 and H1+H4′**. For the sake of clarity only the central GC–radical adduct and the surrounding water molecules are displayed as balls and sticks, while the rest of the system is shown has wireframe.

In addition to the optimal geometry of the canonical GC–radical adducts, we have computed the energetic profiles corresponding both to the transfer of protons H1 and H4, directly exchanged between the basis, and to the tautomeric equilibria arising from the movement of the H4′ (see Figure 1). The latter equilibrium implies a water-assisted mechanism in which the surrounding water molecules catalyze the process by accepting and donating protons (see Ref. Jacquemin et al., 2014 for details). The optimized rare tautomeric structures are shown in Figure 5, and their relative energies and equilibrium constant are given in Table 2. We should note that all the attempts tried to optimize products arising from a single-PT reaction, that is, to optimize rare tautomers where only one proton (H1, H4 or H4′) is shifted from its position, failed as the geometry optimization quickly restored the original canonical structure. However, we were able to localize the products for H1+H4 and H1+H4′ double-PT with both O_2_N–C8(G) and ONO–C8(G) adducts, which structures are shown in Figure 5. As discussed above, the O_2_N–C8(G) binding mode results in more acidic H4 and H1 protons, compared to the ONO–C8(G) adduct, eventually favouring the tautomeric equilibria by ca. 3 kcal.mol^−1^. The associated equilibrium constants (K_eq_) can be estimated from the relative energies obtained using the expression

(1) $${\text{K}}_{\text{eq}}=e^{-\Delta E/RT}$$

where *R* is the ideal gas constant and *T* is the temperature (298.15 K). K_eq_ provides a measure of the mutation frequency, which can be directly compared to the computed value for the radical-free DNA. Taking the undamaged-DNA as a reference for the impact of H1+H4 and H1+H4′ reactions, one notices that the latter is unlikely to occur due to the low K_eq_ values, which are of the order of 10^−16^ and 10^−13^ for the O_2_N–C8(G) and ONO–C8(G) adducts, respectively. However, the energies listed in Table 2 hint that the O_2_N–C8(G) adduct stabilizes the H1+H4 mutation by ca. 2 kcal.mol^−1^, slightly shifting the equilibrium toward the rare tautomeric form (K_eq_ = 7.27 × 10^−08^). Despite of such a difference, all rare tautomers are in the range of observed frequency for the spontaneous mutation, experimentally measured as ranging from 10^−8^ to 10^−10^ (Topal and Fresco, 1976). Interestingly, there is a certain dissimilarity between NO_2_ and OH. The latter clearly shifts the PT reactions toward the rare tautomeric forms of the GC base pair, and its degenerative effects are therefore governed by the transition states along the PT reactions, which in turn determines their lifetimes (Zhang and Eriksson, 2007; Cerón-Carrasco and Jacquemin, 2012). In contrast, NO_2_ does not affect the PT reactions, so it is not necessary to compute the transition states to conclude that they make no impact in the global mutation when present in DNA during cell replication.

**Table 2:** **Relative energy (ΔE/kcal.mol^−1^) and equilibrium constants (K_eq_) at 298 K calculated for the H1+H4 and H1+H4′ tautomeric equilibria of undamaged-DNA, O_2_N–C8(G) and ONO–C8(G) adducts**.

**Adduct**	**Undamaged-DNA**		**ONO–C8(G)**		**O_2_N–C8(G)**	
	**ΔE**	**K_eq_**	**ΔE**	**K_eq_**	**ΔE**	**K_eq_**
Canonic	0.00		0.00		0.00	
H1+H4	11.23	5.75 × 10^−09^	12.96	3.11 × 10^−10^	9.73	7.27 × 10^−08^
H1+H4′	17.37	1.80 × 10^−13^	20.71	6.50 × 10^−16^	17.48	1.52 × 10^−13^

**Figure 6:**
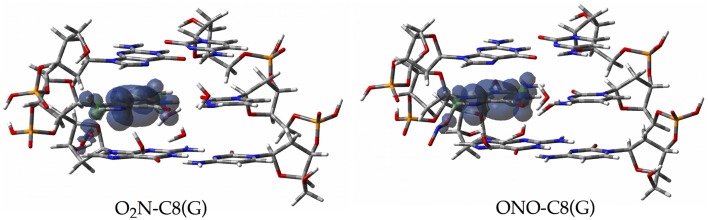
**Spin density distributions of the canonical radical adducts (isovalue = 0.002 a.u.) computed at M06-2X/6-311G(d,p) level for the three base pairs DNA fragment**.

Since the canonical O2N-C8(G) and ONO-C8(G) adducts are shown to be the most stable structures at an early stage of the radical attack, we decided to compute the spin densities surfaces for both forms to determine the localisation of the unpaired electron. Such calculations were performed by single-point full QM calculations at the M06-2X/6-311G(d,p) level. As illustrated in Figure 6, the spin density is distributed in the guanine as well as in the radical, in agreement with previous results obtained for the OH radical (Cerón-Carrasco and Jacquemin, 2012). This finding indicates that the subsequent mutagenic steps will be take place at this region rather than at further sugar-phosphate backbones moieties or at other DNA basis. It becomes therefore necessary to perform additional dynamics calculations with larger models (Loos et al., 2009; Ambrosek et al., 2010; Cauët et al., 2010; Dupont et al., 2011; Garrec et al., 2012; Cerón-Carrasco et al., 2013; Dupont et al., 2013; Patel et al., 2013) for the O_2_N–C8(G) and ONO–C8(G) adducts to further investigate the damage mechanism initiated by GC-NO_2_ structures. Our results indicate that such adducts should evolve by side mechanisms different than PT between guanine an cytosine, e.g., deprotonation to the solvent (Agnihotri and Mishra, 2011).

## 4. Concluding remarks

We have performed theoretical calculations to determine the reaction mechanism between the NO_2_ radical and the neutral closed-shell guanine–cytosine base pair. The reported data demonstrate that the carbon C8 of the guanine moiety is the preferential site to form the GC-radical adduct. The NO_2_ radical equally reacts with the single GC base pair through either its nitrogen or its oxygen atom, with the latter yielding the most stable adduct when a more realistic DNA-embedded model is used. This finding confirms the importance of using a well-balanced chemical model to obtain valuable conclusions. For the radical addition to DNA, the π-stacking interaction not only constrain the planarity of the attacked base pair, but also accommodates better the ONO– binding mode compared to its O_2_N– counterpart. We have assessed the possible evolution of these adducts through proton transfer reactions between guanine and cytosine basis, which is one of the sources of genetic errors in DNA. The associated equilibrium constants lie in the same range as the one observed for spontaneous mutation. We conclude that the initial ONO–C8(G) does not promote rare tautomeric forms by proton exchange, at least not at a rate larger than that in undamaged DNA. The NO_2_-induced genetic damage is expectedly to be initiated by other side reactions.

### Conflict of interest statement

The authors declare that the research was conducted in the absence of any commercial or financial relationships that could be construed as a potential conflict of interest.
